# *rac*-2-{[1-(1-Adamant­yl)eth­yl]imino­meth­yl}-5-meth­oxy­phenol

**DOI:** 10.1107/S1600536811030522

**Published:** 2011-08-02

**Authors:** Xu-Dong Jin, Hai-Bo Wang, Yue-Hong Jin

**Affiliations:** a College of Chemistry, Liaoning University, Shenyang 110036, People’s Republic of China; b Liaoning Provincial Institute of Measurement, Shenyang 110004, People’s Republic of China

## Abstract

A novel Schiff base compound, C_20_H_27_NO_2_, was obtained by a condensation of rimantadine and 2-hy­droxy-4-meth­oxy­benzaldehyde. An intra­molecular O—H⋯N hydrogen bond supports the phenol–imine tautomeric form. The adamantane and imino­methyl-4-meth­oxy­phenol units are arranged in a folded conformation [C—N—C—C torsion angle = 110.9 (3)°]. In the crystal, highly hydro­phobic adamantane moieties are inserted between the imino­methyl-4-meth­oxy­phenol units in a sandwich-like arrangement along the *c* axis.

## Related literature

For the synthesis of 2-((1-(1-adamant­yl)eth­yl)imino­meth­yl)-3-meth­oxy­phenol, see: Shi *et al.* (2006 ▶). For related structures, see: Zhao *et al.* (2005 ▶). For amantadine derivatives, see: Jiang *et al.* (2011 ▶); Jin *et al.* (2011 ▶); Keyser *et al.* (2000 ▶).
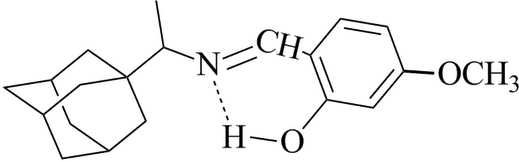


## Experimental

### 

#### Crystal data

C_20_H_27_NO_2_
*M* *_r_* = 313.43
Monoclinic, 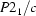

*a* = 9.9656 (12) Å
*b* = 16.1791 (17) Å
*c* = 11.6239 (13) Å
β = 113.575 (1)°
*V* = 1717.7 (3) Å^3^
*Z* = 4
Mo *K*α radiation
μ = 0.08 mm^−1^
*T* = 298 K
0.50 × 0.47 × 0.46 mm

#### Data collection

Bruker SMART CCD area-detector diffractometer
Absorption correction: multi-scan (*SADABS*; Sheldrick, 1996 ▶) *T*_min_ = 0.962, *T*_max_ = 0.965
8451 measured reflections
3016 independent reflections
1794 reflections with *I* > 2σ(*I*)
*R*_int_ = 0.032

#### Refinement

*R*[*F* ^2^ > 2σ(*F* ^2^)] = 0.046
*wR*(*F* ^2^) = 0.137
*S* = 1.06
3016 reflections
210 parameters
H-atom parameters constrained
Δρ_max_ = 0.15 e Å^−3^
Δρ_min_ = −0.18 e Å^−3^

### 

Data collection: *SMART* (Bruker, 2002 ▶); cell refinement: *SAINT* (Bruker, 2002 ▶); data reduction: *SAINT*; program(s) used to solve structure: *SHELXS97* (Sheldrick, 2008 ▶); program(s) used to refine structure: *SHELXL97* (Sheldrick, 2008 ▶); molecular graphics: *SHELXTL* (Sheldrick, 2008 ▶); software used to prepare material for publication: *SHELXTL*.

## Supplementary Material

Crystal structure: contains datablock(s) I, global. DOI: 10.1107/S1600536811030522/kp2341sup1.cif

Supplementary material file. DOI: 10.1107/S1600536811030522/kp2341Isup2.cdx

Structure factors: contains datablock(s) I. DOI: 10.1107/S1600536811030522/kp2341Isup3.hkl

Supplementary material file. DOI: 10.1107/S1600536811030522/kp2341Isup4.cml

Additional supplementary materials:  crystallographic information; 3D view; checkCIF report

## Figures and Tables

**Table 1:** Hydrogen-bond geometry (Å, °)

*D*—H⋯*A*	*D*—H	H⋯*A*	*D*⋯*A*	*D*—H⋯*A*
O1—H1⋯N1	0.82	1.82	2.556 (3)	148

**Fig. 1.**
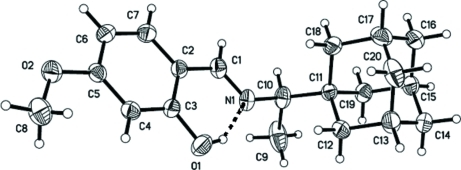
The molecular structure of the title compound with the atomic numbering scheme. Displacement ellipsoids are drawn at the 30% probability level. Dashed lines indicate an intramolecular hydrogen bond.

**Fig. 2.**
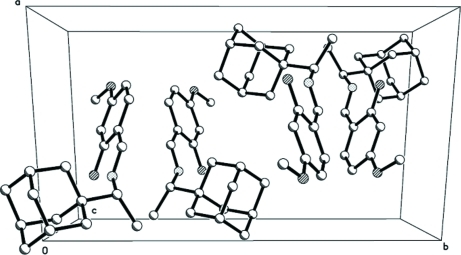
A view of a packing of the title compound in sandwich -like fashion along the c axis.

**Fig. 3.**
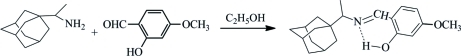
The formation of the title compound.

## supplementary crystallographic information

### Comment

There has been a considerable interest in compounds with adamantane attached to Schiff bases due to their biological activity. The title compound crystallises in the form of phenol-imine tautomer (Fig. 1) with O1—C3 bond length of 1.335 (3) Å. The N1=C10 bond length of 1.273 (3) Å is close to the value described by Shi *et al.* for N=C bond (1.263 (5) Å). Bond length of O2—C8 is 1.421 (3) Å. The torsion angle of C8—O2—C5—C4 is 6.6 (3)°. A strong O1—H···N1 intramolecular hydrogen bond stabilises the phenol-imine tautomer. A six-membered ring is formed by this hydrogen bond with O1···H1 distance of 0.820 Å and H1···N1 distance of 1.820 Å. The distance between N1 and O1 atom is 2.556 Å. The hydrogen bond angle O1—H1···N1 is 148.63°.

### Experimental

A mixture of rimantadine (0.54 g, 3.0 mmol) and 2-hydroxy-4-methoxybenzaldehyde (0.46 g, 3.0 mmol) in anhydrous alcohol (30 mL) was stirred and refluxed for *ca* 4 h. Then the solution was concentrated and left to stand at room temperature. Yellow crystals suitable for X-ray analysis were obtained by slow evaporation of a solvent in a few days.

### Refinement

Hydrogen atoms attached to C atoms were placed in idealized positions with isotropic thermal parameters fixed 1.2 times the value of the attached atom. The H atom attached to O atom was positioned geometrically and refined using a riding model with O—H = 0.82 Å and U_iso_(H) = 1.5U_eq_(O).

### Crystal data

**Table:**

C_20_H_27_NO_2_	*F*(000) = 680
*M**_r_* = 313.43	*D*_x_ = 1.212 Mg m^−^^3^
Monoclinic, *P*2_1_/*c*	Melting point: 376.2 K
Hall symbol: -P 2ybc	Mo *K*α radiation, λ = 0.71073 Å
*a* = 9.9656 (12) Å	Cell parameters from 2307 reflections
*b* = 16.1791 (17) Å	θ = 2.3–25.2°
*c* = 11.6239 (13) Å	µ = 0.08 mm^−^^1^
β = 113.575 (1)°	*T* = 298 K
*V* = 1717.7 (3) Å^3^	Block, yellow
*Z* = 4	0.50 × 0.47 × 0.46 mm

### Data collection

**Table:**

Bruker SMART CCD area-detector diffractometer	3016 independent reflections
Radiation source: fine-focus sealed tube	1794 reflections with *I* > 2σ(*I*)
graphite	*R*_int_ = 0.032
φ and ω scans	θ_max_ = 25.0°, θ_min_ = 2.3°
Absorption correction: multi-scan (*SADABS*; Sheldrick, 1996)	*h* = −11→6
*T*_min_ = 0.962, *T*_max_ = 0.965	*k* = −18→19
8451 measured reflections	*l* = −13→13

### Refinement

**Table:**

Refinement on *F*^2^	Primary atom site location: structure-invariant direct methods
Least-squares matrix: full	Secondary atom site location: difference Fourier map
*R*[*F*^2^ > 2σ(*F*^2^)] = 0.046	Hydrogen site location: inferred from neighbouring sites
*wR*(*F*^2^) = 0.137	H-atom parameters constrained
*S* = 1.06	*w* = 1/[σ^2^(*F*_o_^2^) + (0.0447*P*)^2^ + 0.732*P*] where *P* = (*F*_o_^2^ + 2*F*_c_^2^)/3
3016 reflections	(Δ/σ)_max_ < 0.001
210 parameters	Δρ_max_ = 0.15 e Å^−^^3^
0 restraints	Δρ_min_ = −0.18 e Å^−^^3^

### Special details

**Table:**

Geometry. All e.s.d.'s (except the e.s.d. in the dihedral angle between two l.s. planes) are estimated using the full covariance matrix. The cell e.s.d.'s are taken into account individually in the estimation of e.s.d.'s in distances, angles and torsion angles; correlations between e.s.d.'s in cell parameters are only used when they are defined by crystal symmetry. An approximate (isotropic) treatment of cell e.s.d.'s is used for estimating e.s.d.'s involving l.s. planes.
Refinement. Refinement of *F*^2^ against ALL reflections. The weighted *R*-factor *wR* and goodness of fit *S* are based on *F*^2^, conventional *R*-factors *R* are based on *F*, with *F* set to zero for negative *F*^2^. The threshold expression of *F*^2^ > σ(*F*^2^) is used only for calculating *R*-factors(gt) *etc*. and is not relevant to the choice of reflections for refinement. *R*-factors based on *F*^2^ are statistically about twice as large as those based on *F*, and *R*- factors based on ALL data will be even larger.

### Fractional atomic coordinates and isotropic or equivalent isotropic displacement parameters (Å^2^)

**Table:**

	*x*	*y*	*z*	*U*_iso_*/*U*_eq_	
N1	0.7126 (2)	0.67451 (12)	0.40697 (18)	0.0536 (6)	
O1	0.71762 (19)	0.61250 (12)	0.20647 (17)	0.0695 (6)	
H1	0.7474	0.6253	0.2808	0.104*	
O2	0.29257 (19)	0.64043 (11)	−0.17264 (15)	0.0613 (5)	
C1	0.5815 (3)	0.69731 (14)	0.3429 (2)	0.0512 (6)	
H1A	0.5313	0.7255	0.3832	0.061*	
C2	0.5078 (3)	0.68105 (13)	0.2104 (2)	0.0430 (6)	
C3	0.5797 (3)	0.63829 (14)	0.1467 (2)	0.0457 (6)	
C4	0.5086 (3)	0.62210 (15)	0.0188 (2)	0.0482 (6)	
H4	0.5559	0.5922	−0.0223	0.058*	
C5	0.3691 (3)	0.65031 (14)	−0.0462 (2)	0.0459 (6)	
C6	0.2963 (3)	0.69355 (16)	0.0147 (2)	0.0547 (7)	
H6	0.2016	0.7129	−0.0298	0.066*	
C7	0.3655 (3)	0.70733 (15)	0.1408 (2)	0.0530 (7)	
H7	0.3155	0.7353	0.1815	0.064*	
C8	0.3556 (3)	0.59070 (19)	−0.2388 (3)	0.0742 (9)	
H8A	0.4444	0.6160	−0.2350	0.111*	
H8B	0.2880	0.5858	−0.3249	0.111*	
H8C	0.3769	0.5368	−0.2013	0.111*	
C9	0.9245 (4)	0.7408 (2)	0.5605 (3)	0.0989 (12)	
H9A	0.9899	0.7055	0.5409	0.148*	
H9B	0.9710	0.7585	0.6464	0.148*	
H9C	0.9008	0.7883	0.5064	0.148*	
C10	0.7852 (3)	0.69376 (16)	0.5414 (2)	0.0572 (7)	
H10	0.7210	0.7305	0.5634	0.069*	
C11	0.8090 (2)	0.61413 (14)	0.6205 (2)	0.0415 (5)	
C12	0.8917 (3)	0.54740 (15)	0.5831 (2)	0.0537 (7)	
H12A	0.9874	0.5681	0.5939	0.064*	
H12B	0.8386	0.5334	0.4953	0.064*	
C13	0.9097 (3)	0.46995 (17)	0.6640 (2)	0.0644 (8)	
H13	0.9618	0.4271	0.6386	0.077*	
C14	0.9970 (3)	0.49280 (17)	0.8023 (2)	0.0635 (8)	
H14A	1.0115	0.4441	0.8545	0.076*	
H14B	1.0923	0.5142	0.8134	0.076*	
C15	0.9132 (3)	0.55750 (16)	0.8406 (2)	0.0538 (7)	
H15	0.9688	0.5721	0.9288	0.065*	
C16	0.7645 (3)	0.52422 (19)	0.8248 (2)	0.0664 (8)	
H16A	0.7765	0.4751	0.8758	0.080*	
H16B	0.7124	0.5653	0.8519	0.080*	
C17	0.6783 (3)	0.50353 (18)	0.6881 (2)	0.0656 (8)	
H17	0.5814	0.4827	0.6767	0.079*	
C18	0.6608 (3)	0.57904 (17)	0.6055 (2)	0.0585 (7)	
H18A	0.6060	0.6211	0.6277	0.070*	
H18B	0.6057	0.5640	0.5185	0.070*	
C19	0.8941 (3)	0.63435 (15)	0.7596 (2)	0.0535 (7)	
H19A	0.9896	0.6563	0.7722	0.064*	
H19B	0.8420	0.6763	0.7850	0.064*	
C20	0.7582 (4)	0.43828 (18)	0.6474 (3)	0.0766 (9)	
H20A	0.7021	0.4243	0.5600	0.092*	
H20B	0.7686	0.3887	0.6971	0.092*	

### Atomic displacement parameters (Å^2^)

**Table:**

	*U*^11^	*U*^22^	*U*^33^	*U*^12^	*U*^13^	*U*^23^
N1	0.0612 (15)	0.0518 (12)	0.0394 (12)	0.0056 (10)	0.0113 (11)	0.0069 (9)
O1	0.0574 (12)	0.0889 (14)	0.0548 (11)	0.0256 (10)	0.0148 (9)	0.0000 (10)
O2	0.0559 (11)	0.0824 (13)	0.0427 (11)	−0.0069 (9)	0.0169 (9)	−0.0063 (9)
C1	0.0656 (18)	0.0438 (14)	0.0444 (15)	0.0057 (12)	0.0223 (14)	0.0034 (11)
C2	0.0485 (15)	0.0403 (13)	0.0411 (13)	0.0035 (11)	0.0189 (12)	0.0038 (10)
C3	0.0444 (15)	0.0448 (14)	0.0464 (15)	0.0050 (11)	0.0166 (12)	0.0076 (11)
C4	0.0522 (16)	0.0524 (15)	0.0450 (14)	0.0041 (12)	0.0247 (13)	−0.0016 (11)
C5	0.0458 (15)	0.0518 (14)	0.0397 (14)	−0.0094 (11)	0.0168 (12)	−0.0006 (11)
C6	0.0412 (15)	0.0697 (17)	0.0499 (16)	0.0056 (12)	0.0147 (13)	0.0020 (13)
C7	0.0522 (16)	0.0591 (16)	0.0510 (16)	0.0080 (12)	0.0242 (13)	−0.0010 (12)
C8	0.089 (2)	0.082 (2)	0.0502 (17)	−0.0053 (17)	0.0263 (16)	−0.0154 (15)
C9	0.127 (3)	0.081 (2)	0.061 (2)	−0.045 (2)	0.008 (2)	0.0146 (17)
C10	0.0701 (19)	0.0519 (15)	0.0391 (14)	0.0037 (13)	0.0109 (13)	−0.0006 (12)
C11	0.0409 (13)	0.0481 (13)	0.0326 (12)	0.0007 (11)	0.0115 (10)	−0.0018 (10)
C12	0.0591 (16)	0.0621 (16)	0.0463 (14)	0.0076 (13)	0.0277 (13)	0.0031 (12)
C13	0.089 (2)	0.0580 (16)	0.0567 (17)	0.0246 (15)	0.0402 (16)	0.0096 (14)
C14	0.0623 (18)	0.0699 (18)	0.0592 (17)	0.0175 (14)	0.0252 (15)	0.0209 (14)
C15	0.0552 (16)	0.0680 (17)	0.0335 (13)	0.0038 (13)	0.0130 (12)	0.0029 (12)
C16	0.0627 (18)	0.091 (2)	0.0517 (16)	0.0056 (15)	0.0293 (14)	0.0127 (15)
C17	0.0522 (17)	0.087 (2)	0.0589 (18)	−0.0181 (15)	0.0230 (15)	0.0021 (15)
C18	0.0434 (15)	0.0805 (19)	0.0457 (15)	0.0005 (13)	0.0116 (12)	0.0027 (13)
C19	0.0570 (16)	0.0582 (16)	0.0390 (14)	0.0021 (13)	0.0128 (12)	−0.0044 (12)
C20	0.107 (3)	0.0621 (19)	0.0566 (18)	−0.0220 (17)	0.0288 (18)	0.0018 (14)

### Geometric parameters (Å, °)

**Table:**

N1—C1	1.273 (3)	C11—C18	1.526 (3)
N1—C10	1.469 (3)	C11—C19	1.531 (3)
O1—C3	1.335 (3)	C12—C13	1.534 (3)
O1—H1	0.8200	C12—H12A	0.9700
O2—C5	1.368 (3)	C12—H12B	0.9700
O2—C8	1.421 (3)	C13—C20	1.532 (4)
C1—C2	1.441 (3)	C13—C14	1.536 (4)
C1—H1A	0.9300	C13—H13	0.9800
C2—C7	1.389 (3)	C14—C15	1.512 (3)
C2—C3	1.402 (3)	C14—H14A	0.9700
C3—C4	1.392 (3)	C14—H14B	0.9700
C4—C5	1.368 (3)	C15—C16	1.518 (4)
C4—H4	0.9300	C15—C19	1.525 (3)
C5—C6	1.388 (3)	C15—H15	0.9800
C6—C7	1.366 (3)	C16—C17	1.511 (4)
C6—H6	0.9300	C16—H16A	0.9700
C7—H7	0.9300	C16—H16B	0.9700
C8—H8A	0.9600	C17—C20	1.508 (4)
C8—H8B	0.9600	C17—C18	1.520 (4)
C8—H8C	0.9600	C17—H17	0.9800
C9—C10	1.520 (4)	C18—H18A	0.9700
C9—H9A	0.9600	C18—H18B	0.9700
C9—H9B	0.9600	C19—H19A	0.9700
C9—H9C	0.9600	C19—H19B	0.9700
C10—C11	1.545 (3)	C20—H20A	0.9700
C10—H10	0.9800	C20—H20B	0.9700
C11—C12	1.523 (3)		
			
C1—N1—C10	121.0 (2)	C11—C12—H12B	109.6
C3—O1—H1	109.5	C13—C12—H12B	109.6
C5—O2—C8	118.1 (2)	H12A—C12—H12B	108.1
N1—C1—C2	122.5 (2)	C20—C13—C12	109.2 (2)
N1—C1—H1A	118.7	C20—C13—C14	109.3 (2)
C2—C1—H1A	118.7	C12—C13—C14	108.9 (2)
C7—C2—C3	117.5 (2)	C20—C13—H13	109.8
C7—C2—C1	122.2 (2)	C12—C13—H13	109.8
C3—C2—C1	120.3 (2)	C14—C13—H13	109.8
O1—C3—C4	118.3 (2)	C15—C14—C13	109.0 (2)
O1—C3—C2	121.1 (2)	C15—C14—H14A	109.9
C4—C3—C2	120.5 (2)	C13—C14—H14A	109.9
C5—C4—C3	119.8 (2)	C15—C14—H14B	109.9
C5—C4—H4	120.1	C13—C14—H14B	109.9
C3—C4—H4	120.1	H14A—C14—H14B	108.3
C4—C5—O2	124.1 (2)	C14—C15—C16	110.3 (2)
C4—C5—C6	120.7 (2)	C14—C15—C19	109.3 (2)
O2—C5—C6	115.2 (2)	C16—C15—C19	109.9 (2)
C7—C6—C5	119.1 (2)	C14—C15—H15	109.1
C7—C6—H6	120.4	C16—C15—H15	109.1
C5—C6—H6	120.4	C19—C15—H15	109.1
C6—C7—C2	122.4 (2)	C17—C16—C15	108.8 (2)
C6—C7—H7	118.8	C17—C16—H16A	109.9
C2—C7—H7	118.8	C15—C16—H16A	109.9
O2—C8—H8A	109.5	C17—C16—H16B	109.9
O2—C8—H8B	109.5	C15—C16—H16B	109.9
H8A—C8—H8B	109.5	H16A—C16—H16B	108.3
O2—C8—H8C	109.5	C20—C17—C16	109.6 (2)
H8A—C8—H8C	109.5	C20—C17—C18	107.9 (2)
H8B—C8—H8C	109.5	C16—C17—C18	111.1 (2)
C10—C9—H9A	109.5	C20—C17—H17	109.4
C10—C9—H9B	109.5	C16—C17—H17	109.4
H9A—C9—H9B	109.5	C18—C17—H17	109.4
C10—C9—H9C	109.5	C17—C18—C11	111.4 (2)
H9A—C9—H9C	109.5	C17—C18—H18A	109.3
H9B—C9—H9C	109.5	C11—C18—H18A	109.3
N1—C10—C9	107.1 (2)	C17—C18—H18B	109.3
N1—C10—C11	110.45 (19)	C11—C18—H18B	109.3
C9—C10—C11	114.6 (2)	H18A—C18—H18B	108.0
N1—C10—H10	108.1	C15—C19—C11	110.9 (2)
C9—C10—H10	108.1	C15—C19—H19A	109.5
C11—C10—H10	108.1	C11—C19—H19A	109.5
C12—C11—C18	108.3 (2)	C15—C19—H19B	109.5
C12—C11—C19	108.55 (19)	C11—C19—H19B	109.5
C18—C11—C19	107.60 (19)	H19A—C19—H19B	108.0
C12—C11—C10	113.13 (19)	C17—C20—C13	110.1 (2)
C18—C11—C10	109.3 (2)	C17—C20—H20A	109.6
C19—C11—C10	109.80 (19)	C13—C20—H20A	109.6
C11—C12—C13	110.36 (19)	C17—C20—H20B	109.6
C11—C12—H12A	109.6	C13—C20—H20B	109.6
C13—C12—H12A	109.6	H20A—C20—H20B	108.2
			
C10—N1—C1—C2	178.7 (2)	C19—C11—C12—C13	58.6 (3)
N1—C1—C2—C7	−178.9 (2)	C10—C11—C12—C13	−179.3 (2)
N1—C1—C2—C3	0.3 (4)	C11—C12—C13—C20	58.7 (3)
C7—C2—C3—O1	178.6 (2)	C11—C12—C13—C14	−60.5 (3)
C1—C2—C3—O1	−0.7 (3)	C20—C13—C14—C15	−58.1 (3)
C7—C2—C3—C4	−1.0 (3)	C12—C13—C14—C15	61.0 (3)
C1—C2—C3—C4	179.7 (2)	C13—C14—C15—C16	60.1 (3)
O1—C3—C4—C5	−177.6 (2)	C13—C14—C15—C19	−60.8 (3)
C2—C3—C4—C5	2.0 (3)	C14—C15—C16—C17	−61.3 (3)
C3—C4—C5—O2	177.3 (2)	C19—C15—C16—C17	59.2 (3)
C3—C4—C5—C6	−1.4 (4)	C15—C16—C17—C20	60.8 (3)
C8—O2—C5—C4	6.6 (3)	C15—C16—C17—C18	−58.3 (3)
C8—O2—C5—C6	−174.7 (2)	C20—C17—C18—C11	−61.4 (3)
C4—C5—C6—C7	−0.3 (4)	C16—C17—C18—C11	58.8 (3)
O2—C5—C6—C7	−179.1 (2)	C12—C11—C18—C17	59.9 (3)
C5—C6—C7—C2	1.3 (4)	C19—C11—C18—C17	−57.2 (3)
C3—C2—C7—C6	−0.7 (4)	C10—C11—C18—C17	−176.4 (2)
C1—C2—C7—C6	178.6 (2)	C14—C15—C19—C11	60.2 (3)
C1—N1—C10—C9	−123.6 (3)	C16—C15—C19—C11	−60.9 (3)
C1—N1—C10—C11	110.9 (3)	C12—C11—C19—C15	−58.4 (3)
N1—C10—C11—C12	54.7 (3)	C18—C11—C19—C15	58.5 (3)
C9—C10—C11—C12	−66.5 (3)	C10—C11—C19—C15	177.4 (2)
N1—C10—C11—C18	−66.1 (3)	C16—C17—C20—C13	−60.2 (3)
C9—C10—C11—C18	172.8 (2)	C18—C17—C20—C13	60.8 (3)
N1—C10—C11—C19	176.1 (2)	C12—C13—C20—C17	−60.3 (3)
C9—C10—C11—C19	55.0 (3)	C14—C13—C20—C17	58.7 (3)
C18—C11—C12—C13	−57.9 (3)		

### Hydrogen-bond geometry (Å, °)

**Table:**

*D*—H···*A*	*D*—H	H···*A*	*D*···*A*	*D*—H···*A*
O1—H1···N1	0.82	1.82	2.556 (3)	148
